# Neural correlates of the sound facilitation effect in the modified Simon task in older adults

**DOI:** 10.3389/fnagi.2023.1207707

**Published:** 2023-08-14

**Authors:** Anna Manelis, Hang Hu, Rachel Miceli, Skye Satz, Marie Schwalbe

**Affiliations:** ^1^Department of Psychiatry, University of Pittsburgh, Pittsburgh, PA, United States; ^2^University of Pittsburgh School of Medicine, Pittsburgh, PA, United States

**Keywords:** older adults, interference resolution, fMRI, sound effect, falls, superior parietal lobule

## Abstract

**Introduction:**

The ability to resolve interference declines with age and is attributed to neurodegeneration and reduced cognitive function and mental alertness in older adults. Our previous study revealed that task-irrelevant but environmentally meaningful sounds improve performance on the modified Simon task in older adults. However, little is known about neural correlates of this sound facilitation effect.

**Methods:**

Twenty right-handed older adults [mean age = 72 (SD = 4), 11 female] participated in the fMRI study. They performed the modified Simon task in which the arrows were presented either in the locations matching the arrow direction (congruent trials) or in the locations mismatching the arrow direction (incongruent trials). A total of 50% of all trials were accompanied by task-irrelevant but environmentally meaningful sounds.

**Results:**

Participants were faster on the trials with concurrent sounds, independently of whether trials were congruent or incongruent. The sound effect was associated with activation in the distributed network of auditory, posterior parietal, frontal, and limbic brain regions. The magnitude of the behavioral facilitation effect due to sound was associated with the changes in activation of the bilateral auditory cortex, cuneal cortex, and occipital fusiform gyrus, precuneus, left superior parietal lobule (SPL) for No Sound vs. Sound trials. These changes were associated with the corresponding changes in reaction time (RT). Older adults with a recent history of falls showed greater activation in the left SPL than those without falls history.

**Conclusion:**

Our findings are consistent with the dedifferentiation hypothesis of cognitive aging. The facilitatory effect of sound could be achieved through recruitment of excessive neural resources, which allows older adults to increase attention and mental alertness during task performance. Considering that the SPL is critical for integration of multisensory information, individuals with slower task responses and those with a history of falls may need to recruit this region more actively than individuals with faster responses and those without a fall history to overcome increased difficulty with interference resolution. Future studies should examine the relationship among activation in the SPL, the effect of sound, and falls history in the individuals who are at heightened risk of falls.

## 1. Introduction

Working memory is a cognitive system critical for on-line information processing and manipulation (Baddeley, 2010). One of the working memory functions is to maintain cognitive control despite interference from task-unrelated stimuli and conditions (Engle and Kane, 2004). Interference resolution plays a vital role in decision making, navigating complex environments, and resolving conflicting demands during multitasking (e.g., visual, and auditory interference during driving). The ability to resolve interference declines with age (Collette et al., 2009; Weeks and Hasher, 2014). Age-related performance reduction is associated with weakened inhibitory control, decreased mental alertness, and slowed processing speed (Hasher et al., 1999; Cepeda et al., 2001; Guerreiro et al., 2010; Klein and Ivanoff, 2011; Qin and Basak, 2020).

Although cognitive performance in older adults could be disrupted by distracting stimuli (Juncos-Rabadán et al., 2008; Guerreiro et al., 2013), in some cases, distracting stimuli may facilitate such performance (Weeks and Hasher, 2014; Schwalbe et al., 2023). In our recent study, younger and older individuals performed a modified Simon task in which right- or left-pointing arrows were shown in the locations that were either congruent or incongruent with the arrow direction. Half of the visual stimuli were presented concurrently with the task-irrelevant but environmentally meaningful sounds (Schwalbe et al., 2023). Consistent with the other studies of older adults that used different versions of Simon task (Van Der Lubbe and Verleger, 2002; Juncos-Rabadán et al., 2008; Kubo-Kawai and Kawai, 2010; Maylor et al., 2011), participants were slower for incongruent vs. congruent trials. Contrary to our hypothesis that sounds would create perceptual interference and thus worsen reaction time (RT) on the task, we found that sound presentation significantly improved RT in both younger and older individuals. The neural underpinnings of such facilitation are poorly understood. In the current fMRI study, we aimed to examine the sound effect in older adults and its relationship to behavioral facilitation related to sound presentation. Considering a high rate and detrimental consequences of falls in older adults (Centers for Disease Control and Prevention, 2021) as well as our recent findings that the cognitive interference was greater in older adults with recent falls than in those without falls (Schwalbe et al., 2023), we also explored how the presence of recent falls is related to brain activation in the modified Simon task.

Previous studies have shown that the anterior cingulate, prefrontal, and parietal cortices are involved in interference resolution. The anterior cingulate cortex plays a role in conflict monitoring and response selection (Milham and Banich, 2005; Kelly et al., 2009), while the prefrontal cortex plays a role in cognitive control and decision-making (Wong et al., 2009; Friedman and Robbins, 2022). The posterior parietal cortex plays a role in attentional control, spatial reasoning, and multisensory integration of visual and auditory stimuli, as well as the coordination of movements in response to sensory input (Zhou et al., 2020; Latimer and Freedman, 2023). Decreased activation of the dorsal lateral prefrontal cortex and increased activation of the anterior cingulate cortex have been associated with decreased alertness, which may cause a reduction in cognitive control and an increase in conflict monitoring (Canales-Johnson et al., 2020; Li et al., 2019).

Processing of auditory stimuli relies on the primary and secondary auditory cortices (Langers et al., 2007; Röhl and Uppenkamp, 2012). Sound processing also plays an important role in cognitive function and may engage various brain areas beyond the auditory cortex (Quinci et al., 2022). For example, it was shown that sounds can concurrently activate the visual cortex and improve performance on visual tasks (Feng et al., 2014). In addition, the studies of adaptation to sound repetition indicate that the medial parietal cortex, thalamus, caudate nucleus, and medial occipital cortices could be involved in sound processing (Grady et al., 2011).

Based on this previous research, we hypothesized that performance on incongruent, compared to congruent, trials would be supported by the anterior cingulate, prefrontal, and parietal cortices, while performance on the sound trials would be supported by auditory cortices. The findings from our study may help differentiate between the two prominent hypothesis of cognitive aging: neural compensation and dedifferentiation (McDonough et al., 2022). If a smaller behavioral cost of interference resolution is related to a greater increase in task-related brain activation, this would support the neural compensation hypothesis suggesting that cognitive aging is associated with fronto-parietal increases in activation to compensate for age-related structural and functional decline (Reuter-Lorenz and Cappell, 2008; Cabeza et al., 2018). Alternatively, the findings that a distributed network of cortical and limbic regions is activated during interference trials would be consistent with the dedifferentiation hypothesis suggesting that with age the brain regions’ specialization decreases but the extent of brain activation becomes more widespread (Cabeza and Dennis, 2014; Hülür et al., 2015).

## 2. Materials and methods

### 2.1. Participants

The study was approved by the University of Pittsburgh Institutional Review Board (IRB number STUDY20120072). Written informed consent was obtained from all participants. Twenty right-handed participants between 65 and -80 years of age were recruited from the previous study. That study enrolled participants from the community and the online Pitt + Me and Pepper (IRB number STUDY19090270) registries and did not include a neuroimaging component (Schwalbe et al., 2023). All participants were right-handed, fluent in English, and had premorbid IQ >85 per the National Adult Reading Test (Nelson, 1982). The Montreal Cognitive Assessment (MoCA) cut-off score was 23 to account for participants’ level of education and race (Milani et al., 2018). Exclusion criteria were the standard MRI precautions (e.g., metal in the body and claustrophobia), a history of head injury, neurodevelopmental and neurological disorders, learning disability, current alcohol/drug abuse, and psychiatric disorders except depressive and anxiety disorders. In addition, the data from participants whose head motion inside the scanner [computed using *mriqc* 0.15.1 (Esteban et al., 2017)] exceeded the mean framewise displacement of 0.5 mm (Power et al., 2012) and those whose accuracy was below 75% were excluded from the data analyses. Considering the participants’ age, the medications for high blood pressure or cholesterol were not exclusion criteria.

### 2.2. Demographics, cognitive, and neurological assessments

Information about general demographics, health, and current medications was collected through intake interviews and self-reports. A basic neurological examination was administered by a trained team member to screen for possible neurological deficits. The MoCA (Nasreddine et al., 2005) was used to assess general cognitive functioning across the core domains of cognition. Visual acuity was assessed with the Snellen test. Participants reported fall history for the past year.

### 2.3. Behavioral assessments

**FIGURE 1:**
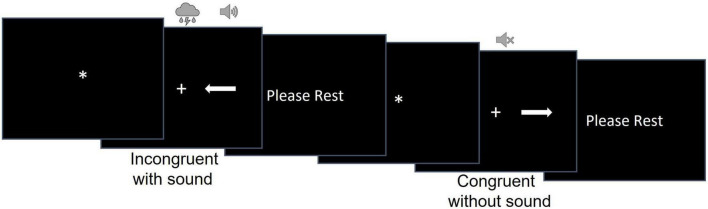
The design of the modified Simon task.

We used a modified version of the Simon task (Schwalbe et al., 2023) to examine participants’ ability to resolve cognitive, perceptual, and combined cognitive and perceptual interference (Figure 1). During this task, participants were shown an arrow pointing either to the left or to the right on the screen. The arrow could be shown on the left or right side of the screen, equidistant from the fixation cross. Participants were instructed to press the response button on the 5-button MRI compatible response system with their right index finger if the arrow was pointing to the right and with their left index figure if the arrow was pointing to the left independently from where on the screen the arrow appeared. Participants were asked to respond as quickly and accurately as possible.

The stimuli were white arrows presented on a black background to the right or to the left of a fixation cross. There were 32 congruent (the arrow’s location and direction matched as when the left-pointing arrow was presented on the left side of the screen) and 32 incongruent (the arrow’s location and direction mismatched as when the left-pointing arrow was presented on the right side of the screen) trials.

The task consisted of 2 runs of 32 trials. Trial duration was randomly determined for each trial and varied between 6,400 and 13,600 ms. Each trial consisted of a fixation star, stimulus presentation, and the rest period [i.e., the inter-trial interval (ITI)]. The fixation star duration ranged between 400 and 3,200 ms in 400 ms increment and was randomly determined for each trial. Stimulus duration was equal to RT but could be no longer than 2,400 ms. The ITI consisted of a “PLEASE REST” screen whose duration was calculated as the difference between the trial duration and the sum of fixation duration and RT for that trial.

A concurrent auditory stimulus started simultaneously with the onset of the visual stimulus (i.e., white arrows) and ended when a participant responded in the trial. Auditory stimuli were natural (e.g., birds chirping, thunderstorm) or man-made (e.g., construction and sirens) sounds and accompanied 50% of congruent and 50% of incongruent trials. The sounds were presented via the MRI compatible Avotec audio system with low profile headphones. To ensure participants could hear the sounds, the sound volume was adjusted to a comfortable level for each participant individually after they were placed inside the scanner. Each run had an equal number of congruent/incongruent and sound/no sound trials. The order of the congruent/incongruent trials with and without sound as well as the trial and fixation durations were randomized for each participant to avoid systematic bias.

### 2.4. Neuroimaging data acquisition

The neuroimaging data were collected at the University of Pittsburgh/UPMC Magnetic Resonance Research Center using a 3T Siemens Prisma scanner with a 64-channel head coil and named according to the ReproIn convention (di Oleggio Castello et al., 2020). The EPI data were collected in the anterior-to-posterior (AP) direction using a multi-band sequence (factor = 8, TR = 800 ms, resolution = 2 × 2 × 2 mm, FOV = 210, TE = 30 ms, flip angle = 52°, 72 slices, 375 volumes). High-resolution T1w images were collected using the MPRAGE sequence (TR = 2,400 ms, resolution = 0.8 × 0.8 × 0.8 mm, 208 slices, FOV = 256, TE = 2.22 ms, flip angle = 8°). Field maps were collected in the AP and posterior-to-anterior (PA) directions using the spin echo sequence (TR = 8,000, resolution = 2 × 2 × 2 mm, FOV = 210, TE = 66 ms, flip angle = 90°, 72 slices).

### 2.5. Data analyses

#### 2.5.1. Behavioral data analysis

The RT values for incorrect responses and those that were outside the 2 IQRs (interquartile range) from the first or third quartile were excluded from the RT analyses. As in our previous study (Schwalbe et al., 2023), two-way mixed-effects models examined the effects of Congruency and Sound on RT (using linear mixed-effect models) and accuracy (using generalized linear mixed-effects models) using the “*lme4*” package in R (Bates et al., 2015). In all models, participants were treated as a random factor and their age, sex, and IQ were used as covariates. For significant effects, the contrasts and means were estimated from the mixed effects models using the “*modebased*” package in R (Makowski et al., 2020).

Considering that all participants were recruited from the previous study in which they completed the same task (albeit outside the scanner), we were able to explore test-retest reliability of the modified Simon task using the inter-class correlation analysis (ICC) function in the “*psych*” package in R (Revelle, 2023).

#### 2.5.2. Neuroimaging data analysis

##### 2.5.2.1. Preprocessing

The DICOM images were converted to a BIDS (Gorgolewski et al., 2016) dataset using *heudiconv version 0.5.4* (Halchenko et al., 2019). Data were examined for quality using *mriqc* 0.15.1 (Esteban et al., 2017) and preprocessed using *fmriprep* 20.1.1 (Esteban et al., 2019). T1w images were skull-stripped, brain surfaces were reconstructed using recon-all (FreeSurfer 6.0.1) (Dale et al., 1999), brain masks were generated, and a reference volume, and its skull-stripped version were generated using *fmriprep* (Esteban et al., 2019). Head-motion parameters were estimated with respect to the BOLD reference before any spatiotemporal filtering using MCFLIRT [FSL 5.0.9 (Jenkinson et al. (2002); RRID:SCR_002823], and applying slice-time correction using *3dTshift* (Cox and Hyde, 1997), (AFNI 20160207; RRID:SCR_005927). Fieldmaps were estimated with *3dQwarp* (Cox and Hyde, 1997) (AFNI 20160207) based on two spin echo images collected with opposing phase-encoding directions (i.e., AP and PA). Based on estimated susceptibility distortion, a corrected EPI (echo-planar imaging) reference was calculated for more accurate co-registration with the anatomical reference. The BOLD reference was co-registered to the T1w reference using *bbregister* (FreeSurfer) (Dale et al., 1999; RRID:SCR_001847) which implements boundary-based registration (Greve and Fischl, 2009). Co-registration was configured with six degrees of freedom. The BOLD time-series were resampled onto the *fsaverage* surfaces (FreeSurfer reconstruction nomenclature) and onto their native space by applying a single, composite transform to correct for head-motion and susceptibility distortions. The BOLD time-series were resampled into standard space, generating a preprocessed BOLD image in MNI152NLin2009cAsym space. Automatic removal of motion artifacts using independent component analysis (ICA-AROMA) (Pruim et al., 2015) was performed on the preprocessed BOLD after removal of non-steady state volumes and spatial smoothing with an isotropic, Gaussian kernel of 6 mm FWHM (full-width half-maximum). After that, global signals within the CSF and WM were extracted and regressed out from preprocessed BOLD data and high-pass temporal filter (90-s cut-off) was applied. All resamplings were performed with a single interpolation step by composing all the pertinent transformations (i.e., head-motion transform matrices, susceptibility distortion correction when available, and co-registrations to anatomical and output spaces). Gridded (volumetric) resamplings were performed using *antsApplyTransforms* (ANTs), configured with Lanczos interpolation to minimize the smoothing effects of other kernels (Lanczos, 1964).

##### 2.5.2.2. Subject-level analysis

Subject-level statistical maps were computed using FSL 6.0.3 installed system-wide on the workstation with GNU/Linux Debian 10 operating system. A hemodynamic response was modeled using a gamma function. A subject-level model included seven explanatory variables: correctly answered congruent trials without sound, correctly answered incongruent trials without sound, correctly answered congruent trials with sound, correctly answered incongruent trials with sound, all trials with incorrect responses, right-hand motor responses, and left-hand motor responses. The motor responses were modeled as the last 200 ms of each trial on which participants responded. The duration for all other conditions was equal to the participants’ RT on that trial. If participants failed to respond within 2.4 s, these trials were considered errors and their duration was modeled as equal to the maximum trial duration of 2.4 s.

The GLM contrasts included comparing all congruent vs. all incongruent trials (i.e., a main effect of congruency), all trials with sound vs. all trials without sound (a main effect of sound), and the interaction between congruency and sound conditions (for exploratory analyses).

##### 2.5.2.3. Group-level analysis

First, we wanted to understand the neural underpinnings for the main effects of sound and stimulus congruency observed in our previous behavioral study (Schwalbe et al., 2023). For this purpose, we contrasted the trials with vs. without sound and congruent vs. incongruent trials using the *swe* (Sandwich Estimator)(Guillaume et al., 2014) approach with 5,000 permutations for non-parametric permutation inference, Threshold-Free Cluster Enhancement (TFCE) correction (Smith and Nichols, 2009), and the FWE-corrected *p*-values threshold set to *p* < 0.01 (0.05/4 = 0.0125) to apply Bonferroni correction for the two contrasts (the effect of sound and the effect of congruency) and two-tailed test (i.e., activation increases and decreases). Age, gender, and IQ were used as covariates in all analyses.

Even though we did not observe the congruency by sound interaction effect on RT and accuracy in the previous study, we still wanted to explore the Congruency-by-Sound interaction effect on brain activation. This exploratory analysis was conducted using the same *swe* approach as describe above with the difference that the FWE-corrected p-values threshold was set to *p* = 0.025 (or 0.05/2) to apply Bonferroni correction for the two-tailed test.

The second exploratory analysis used the mixed effects models to examine whether the RT differences between No Sound and Sound conditions were related to the main and interaction effects between Congruency (Congruent/Incongruent) and No Sound-minus-Sound differences in BOLD percent signal changes in the ROIs determined by the analyses described above. Sex, age, and IQ served as covariates in these models. In addition, to better understand the relationships between brain and behavior, we conducted correlation analyses between participants’ RT and activation in the brain regions showing the Congruency-by-Sound interaction effect for each of the four task conditions.

The third exploratory analysis compared percent signal changes in the brain regions showing Congruency-by-Sound interaction effect in participants with a history of falls during the past 12 months vs. those without such history using a Congruency-by-Sound-by-Falls mixed effects model.

Functional localization was determined using the Harvard-Oxford cortical and subcortical structural atlases and visualized using *fslviewer*. The mean percent signal changes were extracted from the clusters of voxels showing significant differences between the conditions of interest. These values were then used in the follow-up analyses.

## 3. Results

### 3.1. Demographics and behavioral

Twenty individuals [mean (SD) age = 72(4), mean (SD) MoCA = 27.6 (1.8), mean (SD) IQ = 113.9 (5.5), 11 female, 5 participants reported falling within the past 12 months] participated in the study and were included to the data analyses.

**FIGURE 2:**
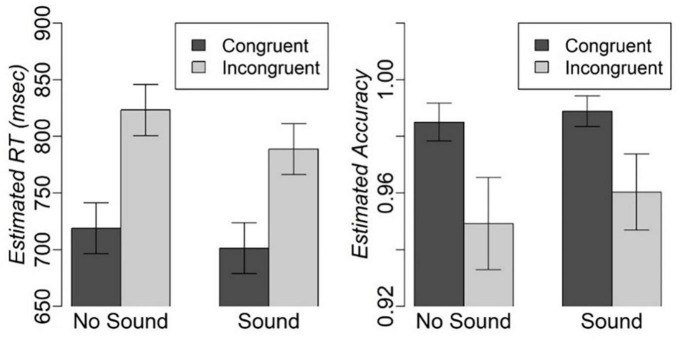
Estimated mean and standard error for RT and accuracy on different types of trials in the modified Simon task.

We excluded 3.4% of RT outliers because they exceeded 2 IQRs. The mixed effect analysis revealed significant main effects of congruency [*F*(1,1156.30) = 121.8, *p* < 0.001] and sound [*F*(1,1156.16) = 9.0, *p* < 0.01], but no Congruency-by-Sound interaction effect on RT. Participants were faster on the congruent compared to incongruent trials [*t*(1156.24) = −11.04, fdr-corrected-*p* < 0.001], and on the trials with concurrent sound compared to the trials without sound [*t*(1156.10) = −3, fdr-corrected-*p* < 0.01] (Figure 2). There was also a significant effect of IQ on RT [*F*(1,16.18) = 11.7, *p* < 0.01]. Individuals with higher IQ responded faster [*t*(16.178) = −3.42, *p* < 0.01]. The analysis of accuracy revealed a significant main effect of congruency with higher accuracy on congruent compared to incongruent trials (*Z*-value = 2.9, *p* < 0.05), but no significant main effect of sound or Congruency-by-Sound interaction effect. Considering that participants were very accurate on the task with over 94% on any type of trial (Figure 2), we will refrain from further discussion of accuracy.

The ICC revealed a moderate-to-high degree of tests-retest reliability between our previous study when participants completed the task outside the scanner (time 1) and the current study in which participants completed the task inside the scanner (time 2). The average measure ICC3k was 0.585 [*F*(19,19) = 2.4, *p* = 0.03] for congruent trials without sound, 0.7 [*F*(19,19) = 3.3, *p* = 0.006] for congruent trials with sound, 0.88 [*F*(19,19) = 8.3, *p* < 0.001] for incongruent trials without sound, and 0.7 [*F*(19,19) = 3.4, *p* = 0.006] for incongruent trials with sound.

### 3.2. Neuroimaging

No participants were excluded from the analyses based on their head motion inside the scanner or behavioral performance accuracy.

#### 3.2.1. The effect of congruency between the arrow direction and location

Our analyses revealed no significant differences between congruent and incongruent trials. No cluster survived the correction for multiple comparisons.

#### 3.2.2. The effect of sound

**FIGURE 3:**
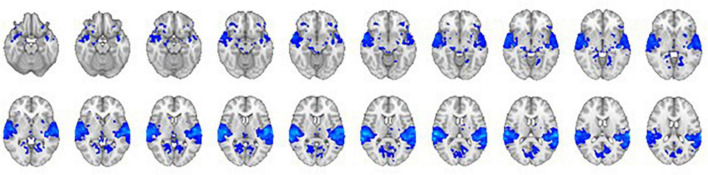
The contrast between trials with sound vs. trials without sound. The right hemisphere is on the left side of the image.

The comparison of the trials with concurrent sound vs. the trials without sound revealed an extended network of regions that showed greater activation for the trials with sound. This network included the bilateral Heschl’s gyrus, superior temporal gyrus, postcentral gyrus, superior parietal lobule (SPL), supramarginal gyrus, thalamus, caudate nucleus, and nucleus accumbens. The activation clusters also extended to the hippocampus as well as the frontal and occipital cortices (Table 1 and Figure 3).

**TABLE 1:** Brain activation for the trails with vs. without sound.

	Region	n-vox	Max *Z*-score	MNI coordinates *X*, *Y*, *Z*
L	Planum temporale, parietal opercular, superior temporal g.	1,5371	12.5	−62, −26, 14
L	Heschl’s g. (includes H1 and H2)		11.8	−52, −12, 6
L	Planum polare, Heschl’s g. (includes H1 and H2)		10.9	−44, −20, −4
L	Superior temporal g.		10.4	−66, −20, 4
R	Planum temporale, parietal opercular, superior temporal g.	5,925	12.2	62, −22, 12
R	Superior temporal g., supramarginal g.		11.2	66, −36, 8
R	Heschl’s g. (includes H1 and H2)		10.7	54, −18, 6
R	Superior temporal g.		9.68	68, −24, 4
R	Planum polare, Heschl’s g. (includes H1 and H2)		9.04	44, −14, −4
L	Thalamus	21	4.94	−6, −12, 10
R	Caudate	19	4.62	10, 18, 8
L	Postcentral g., superior parietal lobule	18	3.87	−22, −40, 74
L	Postcentral g., superior parietal lobule	16	4.3	−18, −40, 66
L	Superior parietal lobule, postcentral g.	16	4.25	−14, −50, 70
R	Nucleus accumbens	13	5.26	6, 12, −6

g., gyrus. The regions without n-vox values are local maxima.

#### 3.2.3. Exploratory analyses

The first exploratory analysis revealed a significant Congruency-by-Sound interaction effect on brain activation in the left SPL, bilateral precuneus, left lateral occipital cortex, cuneal cortex, and occipital fusiform gyrus (Table 2 and Supplementary Figure 1).

**TABLE 2:** The interaction effect of Congruency-by-Sound on brain activation.

	Region	n-vox	Max *Z*-score	MNI coordinates *X*, *Y*, *Z*
L	Occipital fusiform g.	1,026	5.77	−28, −78, −16
R	Lingual g.		5.32	14, −88, −8
R	Occipital pole		4.76	20, −96, −14
L	Lingual g.		4.23	−14, −70, −12
L	Temporal occipital fusiform cortex		4.17	−26, −56, −16
R	Lateral occipital cortex, superior division (LOCsup)	756	5.29	12, −64, 64
R	Precuneus cortex		4.61	6, −60, 56
L	Precuneus cortex		4.23	−4, −42, 48
L	Lingual g.	747	4.5	−22, −84, 2
L	Supracalcarine cortex		4.24	−6, −88, 14
R	Cuneal cortex		3.87	18, −80, 30
R	Lateral occipital cortex, superior division		3.77	16, −84, 24
L	Cuneal cortex		3.71	−8, −84, 28
L	Lateral occipital cortex, superior division (LOCsup)	544	4.72	−38, −72, 22
L	Superior parietal lobule (SPL)	336	4.14	−34, −56, 48
L	Angular g.		4.04	−46, −56, 52
L	Supramarginal g., posterior division		3.55	−42, −42, 42
L	Lateral occipital cortex, superior division		3.5	−44, −56, 60
R	Lateral occipital cortex, superior division	147	5.03	40, −64, 24
R	Lateral occipital cortex, inferior division		4.73	48, −68, 10
R	Precuneus cortex	31	3.82	6, −56, 32
R	Cingulate g, posterior division		3.66	8, −52, 26

g., gyrus. The regions without n-vox values are local maxima.

**FIGURE 4:**
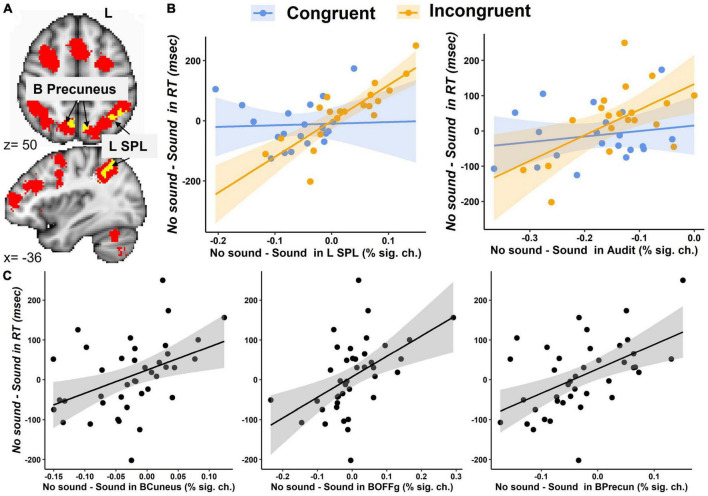
Brain activation during the modified Simon task. **(A)** An overlay between the brain regions showing the Congruency-by-Sound interaction effect (in yellow) and the working memory circuitry derived from the Neurosynth meta-analysis (in red) in the SPL and bilateral precuneus. **(B)** The interaction effect of Congruency and the No Sound-minus-Sound differences in the left SPL and bilateral auditory cortex (Audit). **(C)** The main effect the No Sound-minus-Sound differences in bilateral cuneal cortex (BCuneus), occipital fusiform gyrus (BOFFg), and precuneus (BPrecun) activation on the No Sound-minus-Sound differences in RT.

The brain regions identified in the Congruency-by-Sound interaction analysis could be associated with working memory; therefore, we overlaid the map of the regions revealed in this analysis and the working memory circuitry image that was derived from the 1,091 studies in NeuroSynth meta-analysis (Yarkoni et al., 2011). We found that the left SPL and bilateral precuneus found in our analysis were a part of the working memory circuitry (Figure 4A).

The second exploratory analysis conducted in the bilateral auditory cortex, cuneal cortex, and occipital fusiform gyrus, precuneus, left SPL, and left lateral occipital cortex examined the main effects and interaction of congruency and No Sound-Sound differences in brain activation on the No Sound-Sound differences in RT. A significant interaction effect was found in the left SPL [*F*(1,33) = 11.2, *p* = 0.002], and a marginally significant interaction effect was found in the auditory cortex [*F*(1,33) = 3.5, *p* = 0.07] (Figure 4B). The significant main effects of the No Sound-minus-Sound activation differences on the No Sound-minus-Sound RT differences were found in the bilateral auditory cortex [*F*(1,33) = 5.3, *p* = 0.03], cuneal cortex [*F*(1,33) = 4.9, *p* = 0.03], occipital fusiform gyrus [*F*(1,33) = 9.8, *p* = 0.004], precuneus [*F*(1,33) = 8.2, *p* = 0.007], the left SPL [*F*(1,33) = 7.7, *p* = 0.009] (Figure 4C).

The follow-up correlation analyses between RT and brain activation conducted separately for each task condition revealed a significant positive correlation between RT and brain activation in the left SPL (Congruent – no sound: *r* = 0.58, *p* < 0.01, Congruent – with sound: *r* = 0.51, *p* < 0.05, Incongruent – no sound: *r* = 0.57, *p* < 0.01, Incongruent – with sound: *r* = 0.69, *p* < 0.001). Specifically, lower RT (indicating faster responses) was associated with lower activation in the left SPL (Supplementary Figure 3). The bilateral precuneus positively correlated with RT in the Incongruent – sound (*r* = 0.52, *p* < 0.05), but not any other conditions. The other brain regions showed no significant correlation with RT for either condition.

**FIGURE 5:**
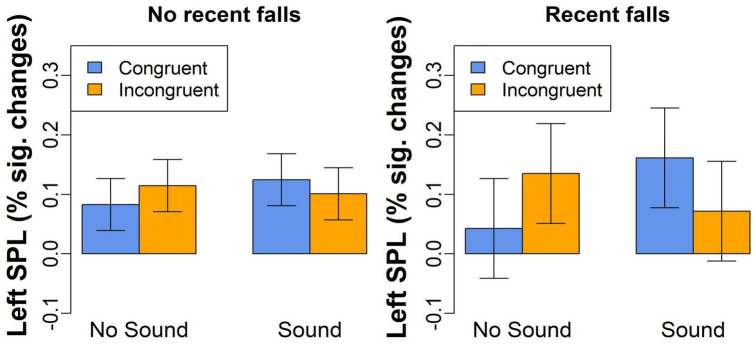
The left SPL activation for congruent and incongruent trials without or without concurrent sound in the individuals with and without a recent history of falls.

The third exploratory analysis conducted on percent signal changes examined whether activation in the brain regions showing Congruency-by-Sound interaction effect was also related to recent falls reported by our participants. We found a significant Congruency-by-Sound-by-Falls interaction effect in the left SPL [*F*(1,54) = 8.5, *p* = 0.005, Figure 5] with the more pronounced increases in brain activation observed in the individuals with recent falls on trials with sound [*t*(54) = 2.5, *p* = 0.015]. No significant main or interaction effects of fall history were revealed in the other brain regions.

The last exploratory analysis examined activation in the anterior cingulate and insular cortices in Congruent/Incongruent and Sound/No Sound conditions. This analysis was conducted to determine whether a lack of significant differences in these ROIs often associated with interference resolution was due to low statistical power that did not permit detecting significant differences in the whole-brain analysis. Given that both of these regions are large and multifunctional, we limited the ROI analyses to the voxels identified through the NeuroSynth (Yarkoni et al., 2011) meta-analysis with the keyword “interference” (uniformity test, fdr-corrected-*p* < 0.01; Supplementary Figure 2). Then, we used the *featquery* tool in FSL to extract percent signal changes from these regions from each participant for each condition of interest (Congruent – no sound, Incongruent – no sound, Congruent – sound, and Incongruent – sound). These percent signal changes were analyzed using the mixed effects model with Congruency, Sound and Congruency-by-Sound interaction as fixed effects and participant as random effect. The analyses revealed no significant main effects of Congruency or Sound, and no significant Congruency-by-Sound interaction (all *p*-values > 0.1).

## 4. Discussion

The aim of the current fMRI study was to understand the neural underpinnings of the sound effect observed in our previous study (Schwalbe et al., 2023). We have replicated the behavioral findings of Schwalbe et al. (2023) and revealed increased activation in a distributed network of regions including the primary and secondary auditory cortices, posterior parietal cortices, thalamus, basal ganglia, hippocampus, and the regions in frontal and occipital cortices for the trials that were accompanied by sounds vs. those that were not.

Considering that the trials with and without sound differed from each other only in the presence of sound, one might expect that brain activation for trials with sound would be localized to the primary and secondary auditory cortices such as Heschl’s gyrus and the planum temporale (Langers et al., 2007; Röhl and Uppenkamp, 2012). Our finding of greater brain activation for sound vs. no-sound trials in the distributed network of fronto-parietal and limbic regions (in addition to auditory cortices) suggests heightened stimulus-driven attention in these trials (Corbetta and Shulman, 2002). The results are also consistent with the dedifferentiation hypothesis of cognitive aging that proposes that neural representations of different types of information change from sparse (in younger individuals) to more distributed (in older individuals) thus leading to reduced specialization of brain regions and more widespread brain activation patterns in older adults (Park et al., 2004; Dennis and Cabeza, 2011; Cabeza and Dennis, 2014; Koen and Rugg, 2019).

While the reduced ability to suppress task-unrelated brain activity may prevent older adults from taking full advantage of relevant brain regions (Logan et al., 2002), it can also benefit task performance by engaging additional brain regions supporting cognitive processing. Also, it can make brain activation patterns for different conditions more similar to each other. For example, several previous studies revealed greater activation for incongruent vs. congruent trials in the frontal, premotor, and SPL regions (Maclin et al., 2001; Wittfoth et al., 2006) and noted involvement of the anterior cingulate and insular cortices in interference resolution (Bunge et al., 2001; Burgess and Braver, 2010). However, contrary to these previous neuroimaging studies and despite finding faster behavioral responses to congruent vs. incongruent trials, we found no significant differences in the brain activation patterns between these trials. To ensure that the lack of findings was not due to low power to detect activation in the whole brain, we conducted the follow up exploratory ROI analyses of percent signal changes in the anterior cingulate and insular cortices. Those analyses also failed to reveal significant main and interaction effects. Although interpreting this lack of the effect is difficult without comparing older individuals with younger counterparts, one possible explanation is the similarity in activation patterns for congruent and incongruent trials with potentially excessive neurocognitive resources allocated to both types of trials. While excessive resource usage reflects neural inefficiency (Reuter-Lorenz et al., 2001; McDonough et al., 2022), it may provide necessary support during cognitive task performance.

Our exploratory analyses revealed that the magnitude of sound-related behavioral facilitation (i.e., the differences in RT for No Sound-minus-Sound conditions) was associated with the changes in activation between No Sound and Sound trials in the bilateral auditory cortex, cuneal cortex, occipital fusiform gyrus, precuneus, and the left SPL. In all these regions, greater differences in RT between No Sound and Sound conditions (i.e., faster RT for Sound vs. No Sound trials) was related to greater differences in activation between No Sound and Sound conditions (i.e., lower brain activation for Sound vs. No Sound trials). These results are inconsistent with the neural compensation hypothesis that predicts greater brain activation for better behavioral performance.

Interestingly, in addition to the main effects, we found a significant BOLD by Congruency interaction in the left SPL with a strong positive relationship between brain and behavior No Sound vs. Sound changes during the Incongruent (more difficult), but not Congruent (easier), condition. Further exploration revealed that the left SPL activation positively correlated with RT in all four task conditions with lower brain activation associated with faster behavioral responses (i.e., lower RT). The SPL plays a critical role for information manipulation and rearrangement in working memory (Koenigs et al., 2009) and usually activates more for more difficult working memory tasks (Owen et al., 2005; Rottschy et al., 2012). The left SPL specifically shows age-related and task-related increases during the tasks requiring cognitive inhibition (Long et al., 2022). Given that performance on the Simon task relies on working memory (Borgmann et al., 2007), slower RT is observed in those older adults for whom sorting out relevant (i.e., the arrow pointing direction) and irrelevant (i.e., the arrow location) features is more difficult.

The SPL is not only linked to working memory but also plays a significant role in visuomotor processing, attention (Alahmadi, 2021), and integration of sensory information from various modalities thus affecting an accurate and coherent representation of body position and movement (Bakker et al., 2008; Desmurget et al., 2009; Wang et al., 2015; Reinhardt et al., 2020). SPL activation is also associated with motor imagery of gait and movement intention during cognitive-motor dual-task conditions (Bakker et al., 2008; Desmurget et al., 2009; Reinhardt et al., 2020). Previous studies have demonstrated that the left posterior parietal cortex plays a role in biasing selection away from salient stimuli in the environment (Mevorach et al., 2006, 2009). Specially, the left SPL, in conjunction with the left pre-supplementary motor area, plays a crucial role in activating the ventral attention network, which responds to task-irrelevant stimuli and helps resolve inferences (Mevorach et al., 2006, 2009; Zhang et al., 2017). In our study, activation in the left SPL was associated with participants’ recent history of falls by showing greater activation for those who fell vs. those who did not. These findings suggest that the individuals with a recent history of falls may have a reduced ability to manipulate upcoming information in working memory. These findings are consistent with previous work indicating that a reduced ability to resolve interference may be a risk factor for falls in older adults (Hausdorff et al., 2005; Nagamatsu et al., 2016; Li et al., 2018; Schwalbe et al., 2023). We would like to notice, however, that although these findings seem plausible for older adults with a history of falls, they are based on a very small sample size (*n* = 5) and, therefore, should be considered as preliminary and need to be reproduced in a larger sample.

The fact that our study participants performed the same modified Simon task twice [in this fMRI study and in the previous behavior-only study (Schwalbe et al., 2023)] allowed us to evaluate the task’s test-retest reliability. The intraclass correlation coefficient was between 0.585 and 0.88 suggesting moderate-to-high reliability that varied depending on the task condition.

One limitation of this study is the small sample size. Future neuroimaging studies should compare larger samples of older adults with and without history of falls to better understand how interference resolution and associated brain responses are related to the past and future history of falls. Considering that stationary broadband environmental noise was shown to benefit balance by potentially serving as an auditory anchor (Lubetzky et al., 2020), future prospective studies should investigate the correlation between participants’ balance, their brain response to cognitive (congruency), and perceptual (sound) interference, and the incidence of falls among older adults, particularly those with different neurological and psychiatric conditions.

## 5. Conclusion

In summary, this neuroimaging study of older adults has replicated the facilitatory effect of task-irrelevant but environmentally meaningful sounds on performance in the modified Simon task. Consistent with the dedifferentiation hypothesis, the sound processing was associated with activation in the distributed network of auditory, posterior parietal, frontal and limbic brain regions suggesting that the effect of facilitation may be achieved through recruitment of multiple neural circuitries some of which are excessive for the task but may allow older adults to increase attention and mental alertness during the task. Our preliminary finding of the relationship between the left SPL activation, RT, and history of falls is indicative of a potential relationship between posterior parietal activation, reduced ability to resolve interference, and falls in older adults.

## Data availability statement

The raw data supporting the conclusions of this article will be made available by the authors, without undue reservation.

## Ethics statement

The study was approved by the University of Pittsburgh Institutional Review Board (IRB number STUDY20120072). The patients/participants provided their written informed consent to participate in this study.

## Author contributions

AM designed the study, curated the study development, evaluated the data quality, analyzed and interpreted the data, and drafted and critically evaluated the manuscript. HH evaluated the data quality, analyzed and interpreted the data, and drafted and critically evaluated the manuscript. RM and SS curated the study development, recruited the participants, acquired the data, evaluated the data quality, and drafted and critically evaluated the manuscript. MS curated the study development, evaluated the data quality, and drafted and critically evaluated the manuscript. All authors had read and approved the final version of the manuscript and agreed to be accountable for all aspects of this work.
