# Supplementary material for: Neural correlates of the sound facilitation effect in the modified Simon task in older adults
Source: Front Aging Neurosci. 2023 Aug 14;15:1207707. doi: 10.3389/fnagi.2023.1207707 (PMC10461020; doi:10.3389/fnagi.2023.1207707)
Supplement: Supplementary file 1 [file Data_Sheet_1.DOCX]

Supplementary Materials

Neural Correlates of the Sound Facilitation Effect in the Modified Simon Task in Older Adults

Manelis A^1*^, Hu H^1^, Miceli R^1^, Satz S^1^, Schwalbe M^2^

*** Correspondence:** Corresponding Author: [anna.manelis@gmail.com](mailto:anna.manelis@gmail.com)

## Supplementary Figures


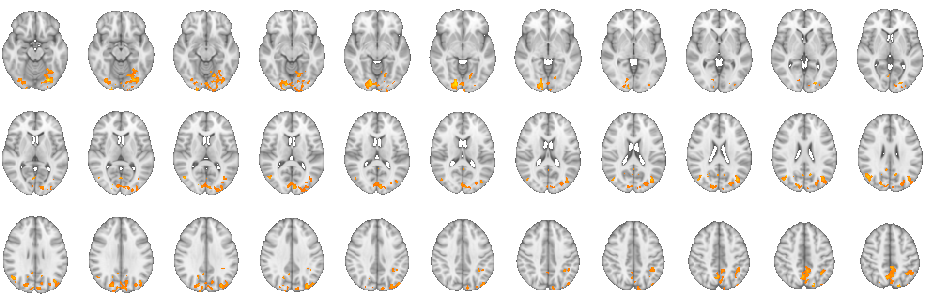


Supplementary Figure S1. Brain regions showing the congruency-by-sound interaction effect. The right hemisphere is on the left.


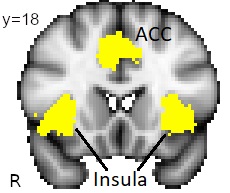


**Supplementary Figure S2.** The masks for the anterior cingulate cortex and bilateral insula ROIs that were used in the exploratory analyses of these regions.


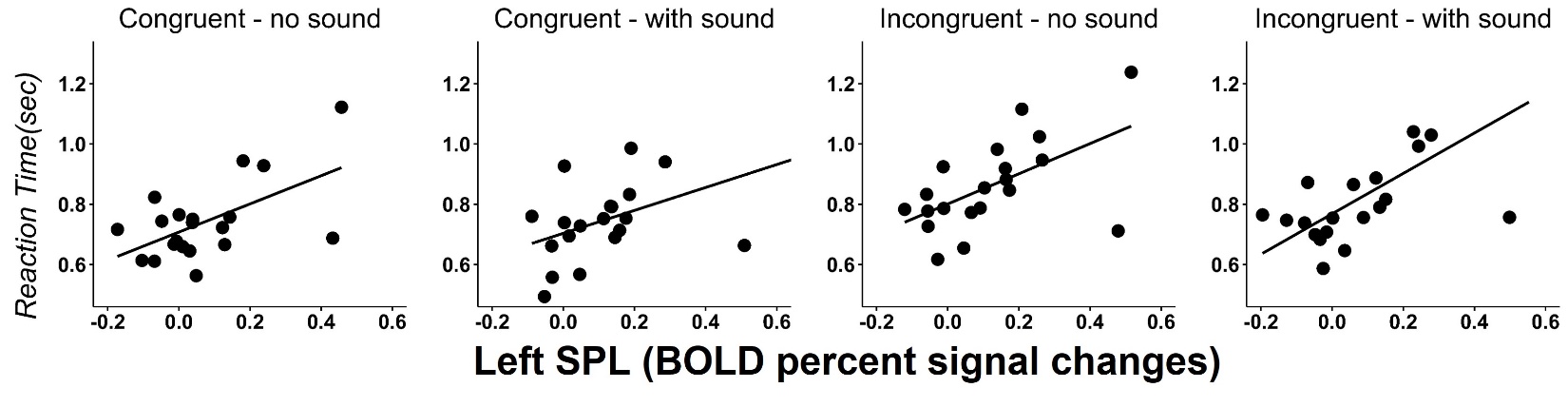


**Supplementary Figure S3**. Correlations between the percent signal changes in the left SPL and RT on the congruent/incongruent trials with and without sound
